# Neurocysticercosis May No Longer Be a Public Health Problem in Mexico

**DOI:** 10.1371/journal.pntd.0000831

**Published:** 2010-12-21

**Authors:** Ana Flisser, Dolores Correa

**Affiliations:** 1 Departamento de Microbiología y Parasitología, Facultad de Medicina, Universidad Nacional Autónoma de México (UNAM), México, DF, México; 2 Laboratorio de Inmunología Experimental, Instituto Nacional de Pediatría, Secretaría de Salud, México, DF, México; University of Queensland, Australia

The Joint Coordinating Board of the Special Programme for Research and Training in Tropical Diseases (TDR) of the World Health Organization in its 25th session in June 2002 endorsed the “Disease Entry/Exit Strategy” within the TDR research portfolio [1]. Here we are using this concept to show how neurocysticercosis (NCC) “entered” as a public health problem and provide data to support why it may no longer be regarded as such in Mexico (“exit”).

**Figure 1:**
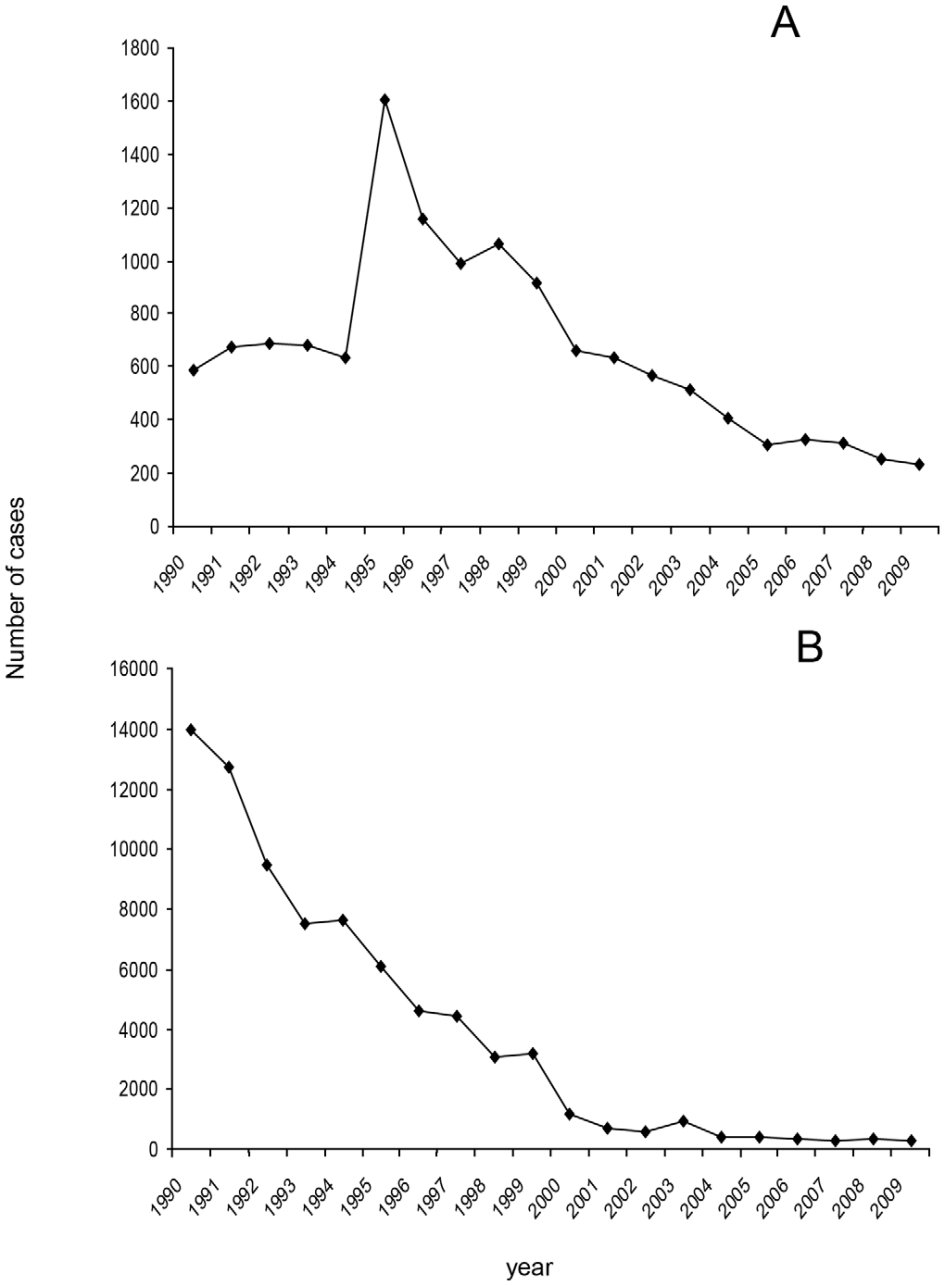
Number of cases per year reported in the official notification system for epidemiologic surveillance (SUIVE) of the Ministry of Health in Mexico. (A) refers to notifications of cysticercosis and (B) to those of taeniosis since 1990.

In 1966, Macias reported that 2.8% of 884 autopsy studies performed between 1947 and 1957 were positive for NCC [2]. This article generated awareness of the disease as a public health problem in Mexico. Five articles concerned with pathological investigations of brains of Mexican people and published in Spanish between 1947 and 1984 reported, on average, infection rates of 2% (reviewed in [3]). The historical evidence presented in these articles is used here as the entry. The exit is proposed here based on the dramatic decrease of cysticercosis and taeniosis frequencies, as obtained from the Information System for Epidemiological Surveillance of the Ministry of Health (SUIVE, Sistema Único de Información para la Vigilancia Epidemiológica) (Figure 1) [4]. A possible explanation for this finding is the establishment of a National Program for the Control of *Taenia solium* that was based on the results published by the Mexican scientific and medical community working on cysticercosis. Another factor of major importance is the general improvement of living conditions in Mexico.

**Figure 2:**
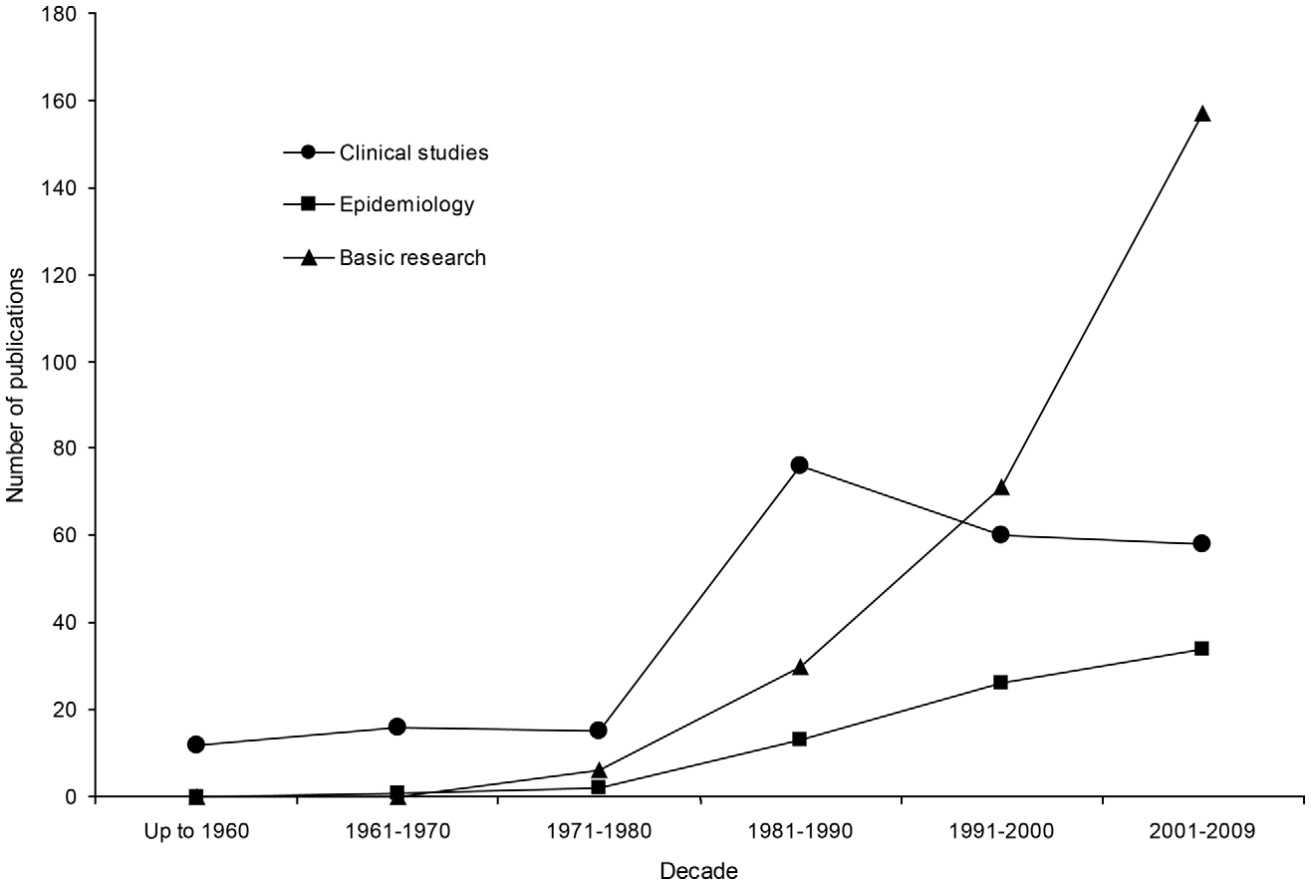
Number of articles on cysticercosis and taeniosis published by Mexican scientists, pooled by decade of publication. Clinical studies include description of cases, drug therapy, immunological and imaging techniques for diagnosis, pathology, and surgery. Epidemiological studies are reports on seroepidemiology and on intervention and control measures. Basic studies refer to immunological findings in patients, experimental models of taeniosis, and research with *Taenia crassiceps*.

Figure 2 shows the number of articles (600) published by Mexican researchers, pooled by decade and main topics (clinical studies, epidemiology, and basic research). The information was obtained from Medline and Scopus databases, except publications from 1889 to 1970, which were retrieved from Mexican journals. Articles related to clinical aspects of NCC peaked in 1981–1990, and then decreased to a plateau; epidemiological studies started in the same decade and have been increasing modestly, while basic biology has had a huge increase since 1991. These trends show a shift in scientific interest from clinical aspects to experimental models and basic biology.

The information generated on imaging and immunological diagnosis, clinical studies, cestocidal treatments, and epidemiological surveys led to the publication of the Official Mexican Guidelines for the Control and Prevention of Taeniosis/Cysticercosis that were published in 1994 [5] and for which an ad hoc working group was organized by the Ministry of Health. The objective of the Guidelines was to establish criteria, strategies, and operative techniques to apply preventive and control measures for taeniosis and human and porcine cysticercosis in the population. The Guidelines are obligatory for the whole Mexican national territory and all public, social, and private health personnel who provide medical attention; professional and technical husbandry personnel, veterinarians, and zoo technicians dedicated to private practice in swine farms; and breeders, owners of swine, and any person involved in moving and commercializing this species. The Guidelines also established that tapeworm carriers should be treated orally with a single dose of praziquantel at 10 mg/kg. Cestocidal treatment is considered imperative since the human tapeworm carrier was identified as the main risk for acquiring NCC [6]. The phenomenon of clustered transmission, where increased transmission occurs in “hotspots” [7], was corroborated in many studies thereafter [3], [8].

At the time of the Guidelines release, hundreds of thousands of pamphlets with basic information targeted to different populations (pig breeders, butchers, cooks, food stand workers, and the general population) were prepared by the Ministry of Health and distributed throughout the country by personnel in charge of promoting *T. solium* control. The Guidelines were revised and published again in the official national newspaper in 2004 [5].

The frequency of human cysticercosis, according to SUIVE (Figure 1A), shows an abrupt increase in 1995 indicating that notifications were associated with the publication and execution of the Guidelines and, therefore, supporting the reliability of their nationwide implementation. Before 1994, between 586 and 691 cases were reported yearly, but in 1995 approximately three times as many cases were reported (1,608) and, since then, notifications have been decreasing and reached 231 cases in 2009. Taeniosis (Figure 1B), in contrast, shows a sharp downward trend from 14,013 cases reported in 1990 to 290 in 2009. Notification is by passive surveillance, since epidemiologists at the municipality level collect the reports from first-level rural clinics up to third-level hospitals and submit them to SUIVE. A study that supports the impact of the National Guidelines on the official notification was the demonstration that self-detection of tapeworm carriers in health centers in an endemic area with around 750,000 inhabitants was successful, since 41 taeniosis patients were recorded in the SUIVE in 1999 as compared to seven in 1998 for that area after an education intervention took place [9].

**Figure 3:**
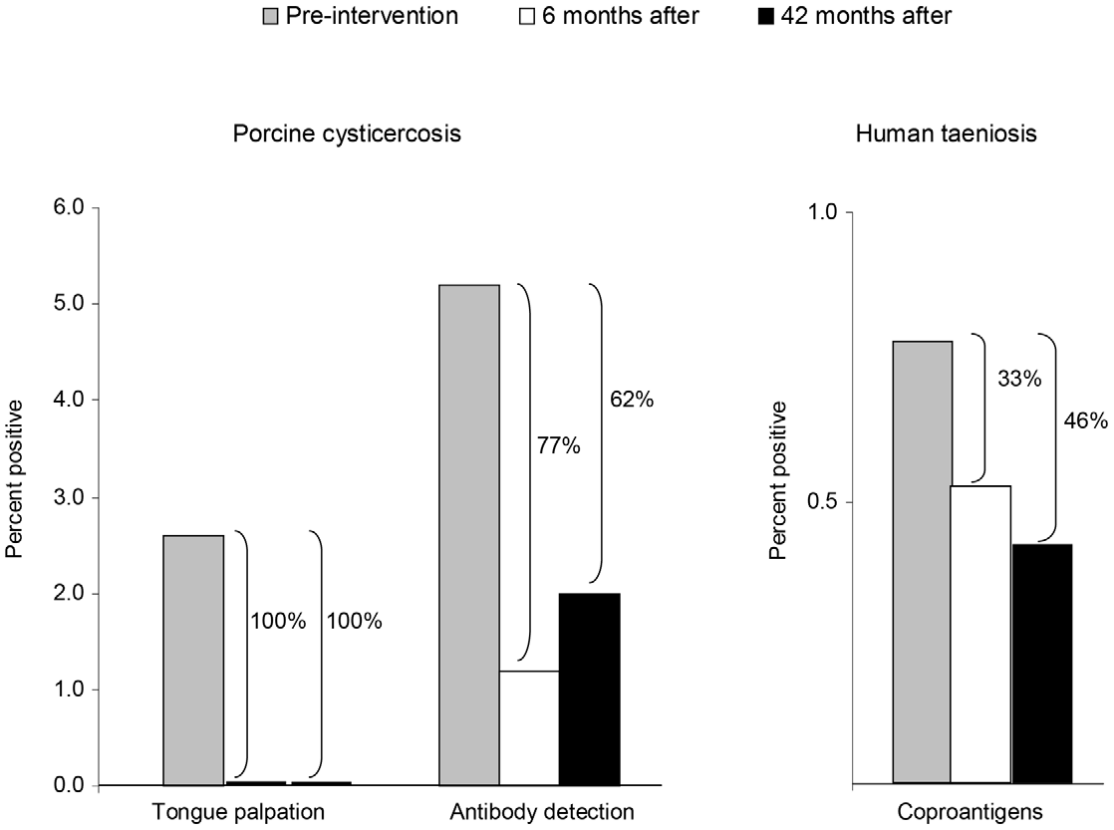
Effect of health education provided to the community of Chalcatzingo, Morelos, Mexico. Evaluation of porcine cysticercosis was measured by tongue palpation and serum antibodies detected by Western blot, while that of human taeniosis was measured by coproantigen detection. Assessment was performed before intervention (*n* = 1,404 for humans and 194 for pigs), 6 months later (*n* = 792 for humans and 165 for pigs), and 42 months later in 1996 (*n* = 605 for humans and 334 for pigs).

Another set of data that supports the usefulness of the national program was obtained from a health education intervention project that took place in Chalcatzingo, state of Morelos, and was implemented by anthropologists and sociologists. In-depth interviews allowed them to identify the knowledge, attitudes, and practices of the inhabitants related to the disease. Natural leaders of the community were trained to transmit information regarding the parasite's life cycle and risks for acquiring NCC and swine cysticercosis [10]. Results were outstanding since after 6 and 42 months no pigs harbored cysticerci in their tongues in comparison to 2.4% before the intervention; also, serum antibodies in pigs decreased approximately 70%. Furthermore, human taeniosis showed a reduction of up to 46% (Figure 3).

**Figure 4:**
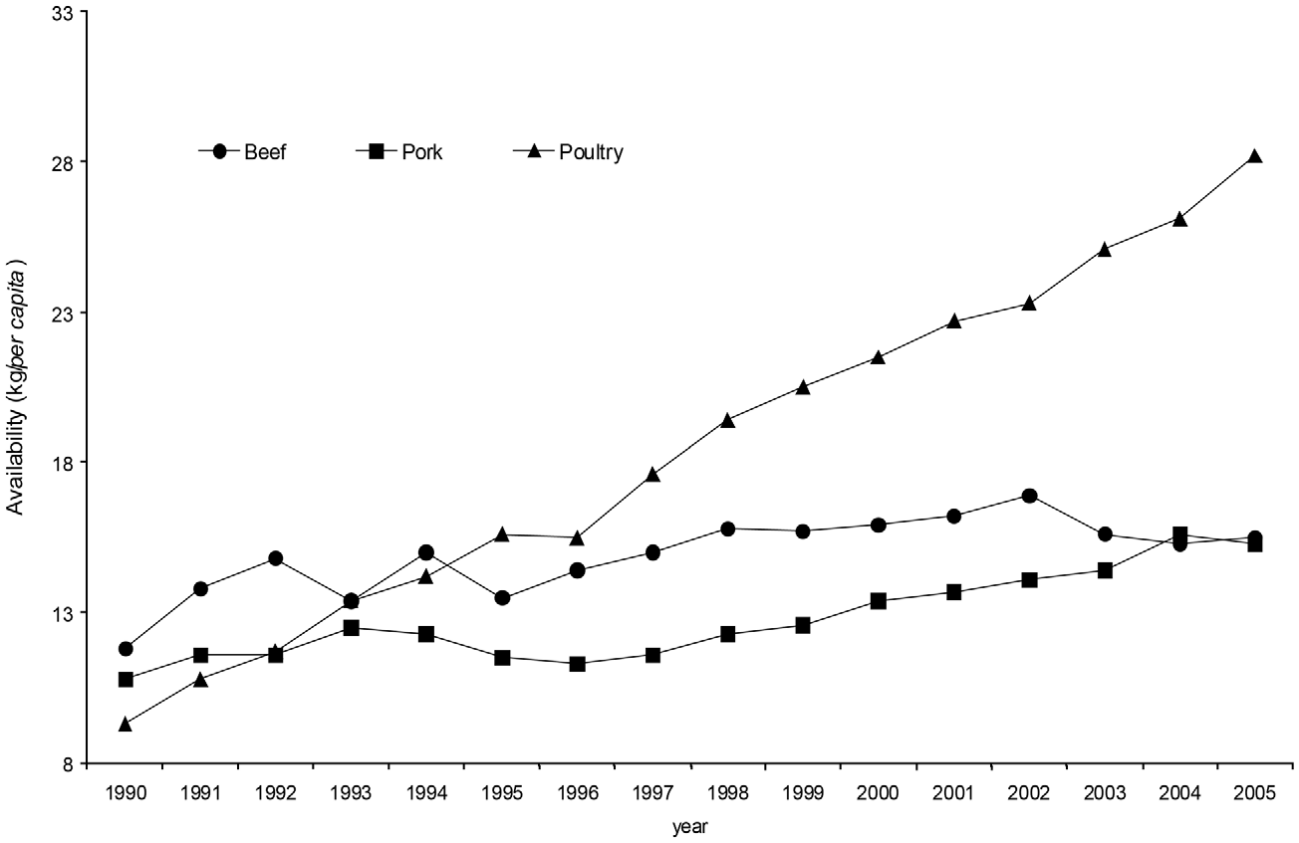
Estimated consumption (kg/capita) of pork, beef, and poultry.

There are no official data on swine cysticercosis in Mexico. Reports on pig slaughter performed in municipal abattoirs or at home indicate that even though more pigs are still being butchered in sub-optimal conditions, those killed in slaughterhouses with federal inspection increased from 30% in 2002 to 40% in 2005. On the other hand, although meat consumption in Mexico is increasing (Figure 4), there has been a shift in the consumption of poultry at the expense of beef and pork [11], and the reduced consumption of red meats could explain why taeniosis has been decreasing.

**Figure 5:**
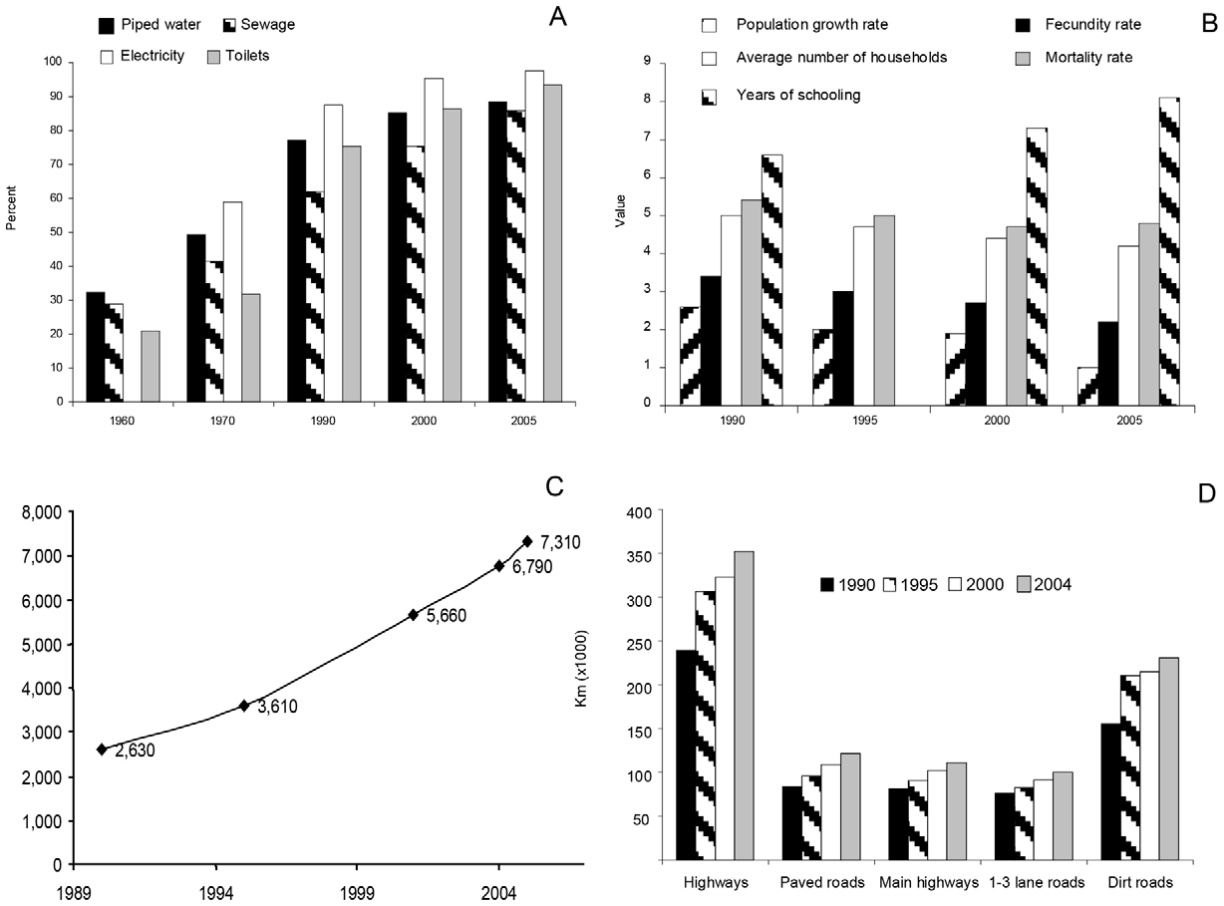
Socioeconomic and health conditions in Mexico that may be related to risk factors for NCC. Percentage of households with the following facilities: piped water, sewage, electricity, and toilets (A); rates per 100,000 inhabitants of population growth, fecundity, and mortality, as well as number of households per home and of schooling years per person (B); per capita income (C); and kilometers of different types of highways and roads built in recent decades (D).

The improvement of socioeconomic and health conditions related to risk factors for NCC in the last decades in Mexico can be seen in Figure 5. Nationwide, a steep increment in household facilities occurred from 1960 to 2005: domiciles with piped water and sewage increased from 31% to 87%, electricity from 59% to 98%, and toilets from 21% to 93% (Figure 5A) [12]. The rate of population growth decreased from 2.6 in 1990 to 1.0 in 2005, fecundity from 3.4 to 2.2, mortality from 5.4 to 4.8, and the number of people in households from 5 to 4.2 in the same period. Interestingly, an increase in the number of schooling years from 6.6 to 8.1 in 15 years took place (Figure 5B) [12]. Income per capita increased from US$2630 in 1990 to US$7310 in 2005 (Figure 5C) [13]. Data on the amount of roads constructed in Mexico in the last 15 years show that dirt roads that allow health and education personnel access into small communities have increased the most as compared to those with one to three lanes, paved roads, or main highways (Figure 5D) [12].

Finally, it should be mentioned that this Viewpoint does not imply that the parasite has been eradicated. As Michael Gemmell stated in the First International Meeting on Cysticercosis, “The stability of the rate of infection depends so much on the egg output into the environment; if toilets are placed and used, you've reduced the infection pressure to zero, a colossal push, like that of Western Europe aimed at increasing standards of living and social hygiene levels. Unstable parasites need only little pushes, but stable ones, like *Taenia ovis* and *Taenia solium*, need big pushes” [14]. Most probably, a stable stage has been achieved in Mexico due to the improvement of socioeconomic conditions, including the huge cholera control campaign that took place between 1991 and 1995, as well as the intensive work and research performed to control NCC. Nevertheless, active surveillance as well as prevention and control measures have to be kept in order to maintain or even improve this situation.
